# Adjudications and self-harm in prisons during COVID-19: three-year longitudinal analysis of the Offender Personality Disorder Pathway in England and Wales

**DOI:** 10.1192/bjo.2025.10883

**Published:** 2025-11-04

**Authors:** Steven M. Gillespie, Andrew Jones, Laura J. Broome, Matthew J. Tonkin, Aisling O’Meara, Carine Lewis, Rachael Dagnall, Shadd Maruna, Jason Davies

**Affiliations:** Department of Primary Care and Mental Health, University of Liverpool, Liverpool, UK; School of Psychology, Liverpool John Moores University, Liverpool, UK; School of Psychology, Swansea University, Swansea, UK; School of Criminology, Sociology and Social Policy, University of Leicester, Leicester, UK; Offender Personality Disorder Pathway, Ministry of Justice, London, UK; School of Psychology, Sports Science and Wellbeing, University of Lincoln, Lincoln, UK; Department of Sociology, Social Policy & Criminology, University of Liverpool, Liverpool, UK

**Keywords:** Conduct disorders, social functioning, personality disorders, psychiatry and law, prison

## Abstract

**Background:**

The effects of pandemic-related restrictions on people in prisons who tend to have multiple complex health needs are not well understood.

**Aims:**

We aimed to measure changes in adjudications and self-harm among people in prisons before and during the pandemic.

**Method:**

We examined effects of time and demographic characteristics on odds and counts of adjudications and self-harm over a three-year period, starting one year before the COVID-19 pandemic, in 861 individuals from 21 Offender Personality Disorder Pathway prison sites.

**Results:**

The odds of adjudicating were lower in people of older age (odds ratio 0.98 (95% CI: 0.96–0.99)), and during COVID-19 year one (odds ratio 0.37 (95% CI: 0.23–0.60)) and year two (odds ratio 0.40 (95% CI: 0.25–0.65)) compared to pre-COVID-19. Being of White ethnicity was associated with increased odds (odds ratio 4.42 (95% CI: 2.06–9.47)) and being older was associated with reduced odds (odds ratio 0.97 (95% CI: 0.95–0.99)) of self-harm. The odds of self-harm were significantly reduced during COVID-19 year two (odds ratio 0.45 (95% CI: 0.26–0.78)), but not during COVID-19 year one (odds ratio 0.68 (95% CI: 0.40–1.14)), compared with the 12 months before COVID-19.

**Conclusions:**

Although adjudications and self-harm were generally lower during the pandemic, younger people showed increased odds of adjudications and self-harm compared with older people, while White people showed increased odds of self-harm compared with people of the global majority. Our findings highlight the importance of considering potential health inequities and environmental effects of lockdowns for people in prisons.

After the coronavirus disease 2019 (COVID-19) was declared a pandemic by the World Health Organization (WHO), on 11 March 2020, there was widespread concern about the potential adverse effects of the pandemic and associated restrictions to reduce transmission of the virus on mental health.^1–3^ Despite an overall increase in mental health symptoms early in the pandemic, a general decline to levels observed before the pandemic has subsequently been reported in longitudinal studies.^4^ Data also suggest that COVID-19 risk mitigation measures did not lead to population-level increases in suicide rates in high-income and upper-middle-income countries in the early months of the pandemic.^5^ Although these results can be viewed as reassuring, there is evidence that the effects of the pandemic exacerbated existing inequalities in mental health and well-being experienced by vulnerable groups, including older people, children/students, low-income populations, people in prison, people with disabilities and people from ethnic, sexual and gender minorities.^6,7^

### COVID-19 in prisons

People in prisons represent a particularly vulnerable group, who have multiple complex health needs and worse health outcomes relative to the general population worldwide.^8^ People in prison are more likely to suffer from mental health problems, including serious mental disorder, personality disorder and drug and alcohol misuse,^9^ while rates of suicide and self-harm are also elevated in prisons globally.^8,9^ Data for England and Wales show that in the 12 months to March 2020, that is, the 12 months immediately prior to the start of the COVID-19 pandemic, rates of self-harm and assaults on other prisoners and staff were all comparatively high compared to earlier years.^10^

### Prison responses to COVID-19

Following the announcement of the first national lockdown in England, prisons moved into ‘command mode’^11^ and operated under the COVID-19 National Framework for Prison Regimes and Services.^11^ Infection control measures included social distancing, limiting the time prisoners spent outside of their cells and restricting movement around the prison. Despite service disruption having the potential to exacerbate poor outcomes for people in prison,^12^ relatively little research has examined the potential adverse consequences of the pandemic and the associated restrictions in this high-risk group.

### Study rationale and purpose

In this study, we examined change in the number of adjudications (that is, formal disciplinary processes where a person is alleged to have broken prison rules, including assaults on prisoners and staff, possession of controlled substances) and incidents of self-harm, over a three-year period using routinely collected data in prisons on the Offender Personality Disorder Pathway (OPDP) residential services in England and Wales. The OPDP is a specialist service in England and Wales for individuals who represent a high-risk of harm to themselves or others, have complex personality difficulties and multiple psychosocial needs,^13,14^ and for men, have committed a violent or sexual offence. Individuals are identified as meeting inclusion criteria via the Offender Assessment System (OASys) and intervention needs are determined via a formulation-based approach or self-referral. This group represented a particularly vulnerable group during the pandemic as these individuals were likely to react more quickly and acutely to environmental and procedural changes resulting from COVID-19. The OPDP residential intervention services have several common standardised key features making it suitable for learning and the extrapolation of results that could be generalised to the wider prison estate. Both adjudications and self-harm represent routinely measured and well-established indicators of both individual mental health and broader institutional stability within prison settings, and our focus on these outcomes allows for direct comparison with official data and enhances the translational value of our findings.

We also examined how the number of adjudications and incidents of self-harm varied with characteristics including gender, age and ethnicity. Prisons are separated by gender, and the demographic, health and criminal characteristics of females in prisons are different to males. Older adults in prison also represent a particularly vulnerable group with unique health, social and custodial needs.^15,16^ These needs may leave older adults particularly vulnerable during periods of shielding to reduce contact with potentially infected others, during times of reduced social visits, disruptions to health care and recreational activities, and increased time spent in cells.^12^

Understanding changes in the number of adjudications and incidents of self-harm before, during and following the pandemic, and the effects of demographic characteristics, represents a valuable learning opportunity to understand how similar restrictions might impact on risk of harm to self and others in the future, and has implications for enhancing policy and practice in preparedness for any future events.

## Method

### Sample

Demographic information (gender, age and ethnicity) and routinely collected data on adjudications and self-harm were shared under licence by the Ministry of Justice. There were 60 OPDP prison sites with a combined capacity of 1338 in 2021. During the pandemic, many non-residential services were unable to operate but residential sites (prison wings) were able to continue in a limited capacity. Data included in this study related to information on 861 individuals in 21 residential OPDP prison sites over a three-year period, from 1 March 2019 to 1 March 2022. Included participants had to be a resident in one of the 21 included sites at the start of the study period. Most participants were White British, male, with an average age of 36.9 years (see Table 1).

**Table 1:** Demographic information from final analytic sample

Variable	Count (%)
Gender	
Male	670 (77.8)
Female	191 (22.2)
Ethnicity	
White	636 (73.9)
Black/African/Caribbean	126 (14.6)
Asian	32 (3.7)
Mixed/Multiple	52 (6.0)
Other	8 (0.9)
Unknown	7 (0.8)
	Mean (s.d.)
Age	36.87 (11.38)

### Outcomes

Data on adjudications included each individual instance of a disciplinary process for an assault by a prisoner within the cohort on a prisoner, staff member or any other person (including attempts), absconding (including attempts), use of threats/abusive and insulting racist words/behaviour, causing damage to any part of a prison, possession of unauthorised articles, disobeying any lawful order, drug/alcohol related offences, being absent from where they should be/present where they should not be, endangering the health/safety of any person, obstructing an officer executing their duty and detaining any person. Over the course of the full study period, 839 of the participants had adjudications recorded against them while they were in one of the prison sites with OPDP services, amounting to 8286 total adjudications for this group. Outcomes from these adjudications vary, including for example ‘proven’, ‘dismissed’, ‘quashed’ or ‘referred to police’. Collectively, 78% (*n* = 6466) were proven, prosecuted or referred to police, while 22% (*n* = 1820) were dismissed or otherwise not pursued (charge not proceeded with 850; charge proved 6307; discharged 186; dismissed 545; police prosecution 6; quashed 6; quashed on appeal 3; referred to police 153; suspended 230). Adjudications were included regardless of plea description (guilty/not guilty) and outcome (e.g. proven or dismissed), offering a broader snapshot of overall adjudications and alleged behaviour during the study period with the large majority of adjudications being proven. Data on self-harm included each individual instance of hanging, self-strangulation, cutting, burning and self-poisoning/overdose/swallowing objects. Over the course of the full study period, 308 participants were recorded as having carried out acts of self-harm, and this amounted to 5928 total acts of self-harm for this group.

### Statistical analysis

Data were divided into three time-periods: Pre-COVID-19 (1 March 2019 to 29 February 2020); first year of COVID-19 (1 March 2020 to 28 February 2021) and second year of COVID-19 (1 March 2021 to 28 February 2022). To be eligible for inclusion in a single time-period, a person had to have been resident in one of the included OPDP prison sites for the duration of that 12-month time-period. All people in the included sites were automatically eligible for inclusion, but if a prisoner was transferred in or out of an OPDP prison site in the middle of a time-period, then they were excluded from the duration of that time-period.

Data were modelled in two ways. First, we modelled each outcome (adjudications or self-harm) as a binary variable, and second as a count variable. To examine the effects of time-period and demographic information (gender: male/female, ethnicity: White/not White, and age: continuous) on the occurrence of adjudications and self-harm as binary variables (yes versus no), we used multilevel binary logistic regressions. Next, we operationalised each outcome as a count variable and analysed only time-points in which at least one adjudication or incidence of self-harm occurred within individuals (i.e. individuals were only included in a time-period if they had at least once instance of an adjudication or self-harm) and fit a multilevel zero-truncated Poisson model to predict the number of occurrences. Zero-truncated Poisson regression is used to model count data for which the value zero cannot occur. Separate binary logistic and zero-truncated Poisson models were calculated for each outcome.

Akaike information criterion (AIC) and Bayesian information criterion (BIC) were used to determine the fit of multi-level models, with significant reductions indicative of better model fit (Akaike, 1974). We fit multilevel models based on two levels, with time-points nested within prisons. The better fit of a two-level model may reflect that individuals did not remain in the OPDP site for the full three-year period (meaning minimum nesting of time-points within individuals) but might also reflect shared characteristics of individuals and practices within the individual prison sites, leading to the non-independence of data. However, findings were similar when using a three-level model, except for ethnicity no longer being a significant predictor of adjudication counts. All analyses were conducted using the ‘lme4’ and ‘glmmTMB’ packages for R using R Studio for macOS (Posit Team, PBC, Boston, MA, USA; http://www.posit.co/).

## Results

### Adjudications (binary)

The analysis included 344 individual participants who were present for at least one full time-period (with 47 individuals present for all three time-periods). These participants contributed a total of 530 individual time-points across the three-year period, with at least one adjudication being reported for 300 time-points. The likelihood of at least one instance of adjudication within any given time-period where a service user was present for the full year was 57% (95% CI: 52–61%). Table 2 (left panel) shows the overall regression model. The regression model explained 13% of the total variance (conditional *R* ^*2*^ = 0.13), with the fixed effects contributing approximately 7% of the variance (marginal *R* ^*2*^ = 0.065). Older age was associated with lower odds of adjudicating, and there were fewer instances of any adjudication during COVID-19 year one (50.0%), and COVID-19 year two (53.5%), compared with the 12 months pre-COVID-19 (69.9%).

**Table 2:** Results of binary (left column) and zero-truncated (right column) regressions predicting adjudications

	Binary adjudication	Count adjudication
Predictor	Odds ratio [95% CI]	Incidence rate [95% CI]
Gender (F)	1.20 [0.53–2.71]	0.82 [0.45–1.49]
Ethnicity (non-White)	0.89 [0.53–1.47]	0.84 [0.73–0.96]*
Age	0.98 [0.96–0.99]*	0.96 [0.95–0.97]*
COVID-19 year 1 (before COVID-19)	0.37 [0.23–0.60]*	0.87 [0.76–1.00]*
COVID-19 year 2 (before COVID-19)	0.40 [0.25–0.65]*	0.77 [0.67–0.88]*

Reference categories are shown in brackets.

*p < 0.05.

### Adjudications (count)

**Fig. 1:**
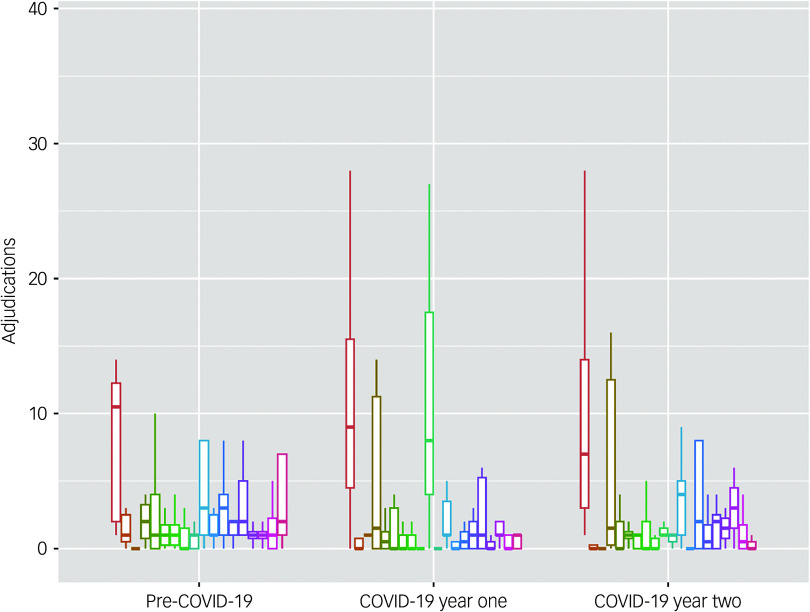
Average number of adjudications per person by prison site (indicated by coloured bars), during each time-period (pre-COVID-19, COVID-19 year one and COVID-19 year two).

Figure 1 shows the average number of adjudications per person by prison site, consistent with the use of a multi-level model, during each time-period (Pre-COVID-19, COVID-19 year one and COVID-19 year two). The analysis consisted of 300 independent observations. Table 2 (right panel) shows the overall regression model. The regression model explained 67% of the total variance (conditional *R* ^*2*^ = 0.67), with the fixed effects contributing approximately 33% of the variance (marginal *R* ^*2*^ = 0.33). Being of White ethnicity (versus not white) or being older were associated with lower adjudication counts. The average count of adjudications per person was lower during COVID-19 year one (mean = 2.49, s.d. = 5.91) and COVID-19 year two (mean = 2.46, s.d. = 5.40) compared with the 12 months before COVID-19 (mean = 3.01, s.d. = 5.50).

### Self-harm (binary)

The analysis included 344 individual participants who were present for at least one full time-period (with 47 individuals present for all three time-periods). These participants contributed a total of 530 individual time-points, across the three-year period, with at least one instance of self-harm being reported for 127 time-points. The likelihood of at least one instance of self-harm within any given time-period where a service user was present for the full year was 24% (95% CI: 20 to 28%). Based on the AIC/BIC model fit statistic, the two-level model of time-points within prisons was most appropriate. Table 3 (left panel) shows the overall regression model. The regression model explained 21% of the total variance (conditional R^*2*^ = 0.21), with the fixed effects contributing approximately 13% of the variance (marginal *R* ^*2*^ = 0.128). Being of White ethnicity was associated with increased odds of self-harm, while being older was associated with reduced odds. There was also a significant reduction in self-harm during COVID-19 year two (19.3%), but not during COVID-19 year one (24.5%), compared with the 12 months before COVID-19 (29.4%).

**Table 3:** Results of binary (left column) and zero-truncated (right column) regressions predicting self-harm

	Binary self-harm	Count self-harm
Predictor	Odds ratio [95% CI]	Incidence rate [95% CI]
Gender (F)	0.48 [0.01–1.18]	0.34 [0.10–1.15]
Ethnicity (non-White)	4.42 [2.06–9.47]*	1.36 [1.03–1.79]*
Age	0.97 [0.95–0.99]*	0.94 [0.93–0.95]*
COVID-19 year one (before COVID-19)	0.68 [0.40–1.14]	1.03 [0.90–1.17]
COVID-19 year two (before COVID-19)	0.45 [0.26–0.78]*	0.61 [0.52–0.71]*

Reference categories are shown in brackets.

* p < 0.05.

### Self-harm (count)

**Fig. 2:**
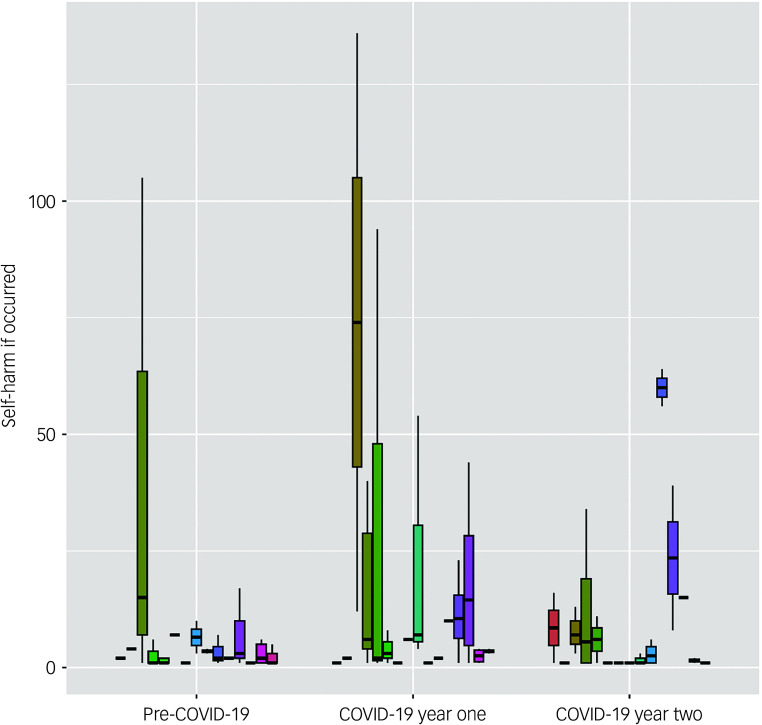
Average number of incidents of self-harm (if > 0) per person by prison site (indicated by coloured bars), during each time-period (pre-COVID-19, COVID-19 year one and COVID-19 year two).

Figure 2 shows the average number of incidents of self-harm per person (not including any time-points in which no self-harm per individual was recorded, which would greatly skew the figures) by prison site, consistent with the use of a multi-level model, during each time-period (Pre-COVID-19, COVID-19 year one and COVID-19 year two). The analysis included 127 independent observations. Table 3 (right panel) shows the overall regression model. The regression model explained 67% of the total variance (conditional *R* ^*2*^ = 0.67), with the fixed effects contributing approximately 33% of the variance (marginal *R* ^*2*^ = 0.33). Being of White ethnicity was associated with higher counts of self-harm, while being older was associated with lower counts. The incidence of self-harm was lower in COVID-19 year two (mean = 1.87, s.d. = 7.73) but not COVID-19 year one (mean = 3.27, s.d. = 13.56), compared with the 12 months before COVID-19 (mean = 3.13, s.d. = 14.01).

## Discussion

In this longitudinal study, we examined changes in the number of adjudications and incidents of self-harm in OPDP prison sites in England and Wales, using routinely collected data across a three-year time-period. We also examined the effects of sociodemographic characteristics, including gender, ethnicity and age, on adjudications and self-harm to aid the identification of potentially vulnerable groups.

Our results showed that increasing age was associated with reduced odds of adjudicating and lower counts of adjudications. These results are generally consistent with work showing that aggressive and antisocial behaviour tends to decline with older age.^17,18^ Being of White ethnicity was also associated with a lower count of adjudications, but not reduced odds, suggesting that White and non-White individuals are equally likely to be initially reported or sanctioned but accumulate fewer adjudications once that threshold is passed. This pattern might reflect differences in ongoing scrutiny or surveillance, behavioural adaptation or systematic bias in responding to White and non-White participants following an initial adjudication. This finding highlights the dual nature of adjudications – that they reflect both individual and institutional behaviour. The Ministry of Justice Race Review 2008^19^ recognises that such differences ‘may be the result of negative stereotyping by some officers… or a lack of understanding of different cultural behaviours, both of which could lead to [Black and minority ethnic] prisoners being treated, at best, differently to White prisoners and, at worst, more harshly and in a manner consistent with discrimination’.

The effect of time showed that both the odds of adjudicating, and the count of adjudications, was significantly reduced during COVID-19 years one and two compared to the 12 months before COVID-19. Several factors should be considered when interpreting these results. First, one of the major restrictions that was introduced to prevent the spread of COVID-19 was to limit the amount of time outside of cells, with most prison sites restricting time spent out of cells to between 30 and 120 minutes per day, over a period of at least 12 months between April 2020 and March 2021.^20^ These restrictions severely limited the time at risk for engaging in behaviour likely to result in an adjudication. For example, the risk of assaults on other prisoners and staff would naturally be much lower, as would the likelihood that a person could be found in a place they should not be/not in a place they should be.

Restrictions on time spent out of cells remained in place throughout COVID-19 year one, and most of COVID-19 year two, with a relaxing of restrictions only beginning to be introduced in February/March 2022. Thus, the pattern of adjudications following the complete relaxing of restrictions cannot be gleaned from these data, but trends for the prison service nationally suggest that rates of prisoner-on-prisoner and prisoner-on-staff assaults have started to steadily return to rates seen before the start of the pandemic.^10^ It will therefore be important to understand the environmental factors that might contribute to adjudications, including prisoner-on-prisoner and staff assaults, following the easing of restrictions. At a policy level, our data show evidence that the longer time spent in cells did not increase the rate of adjudications, but there may be adverse consequences of these restrictions in the short-, medium- and long-term, including for mental health and well-being that were not adequately measured in this study.

As well as lower rates and counts of adjudications, our data also revealed a significant reduction in the odds and counts of self-harm in the second year of COVID-19, and that being of White ethnicity was associated with higher odds, and being younger with lower odds, of self-harm. Similar findings were also observed for count data. Although we found no effects of gender on self-harm, these findings are consistent with a large meta-analysis that reported only a marginal, non-significant effect of gender on self-harm in prisons.^21^ However, our results appear in contrast with the Safety in Custody annual self-harm summary statistics for England and Wales, where the rate of self-harm per 1000 prisoners is higher in females than in males (self-harm incidents per 1000 men: 2020 = 563, 2021 = 557, 2022 = 508; self-harm incidents per 1000 women: 2020 = 3570, 2021 = 3702, 2022 = 5060). It would be interesting in future research to examine what aspects of OPDP, and non-OPDP prison environments may be risk-factors for self-harm in women and in men.^22^ Increased incidence associated with White ethnicity and reduced incidence associated with older age are largely consistent with meta-analytic results,^21^ and are indicative that sociodemographic and environmental risk-factors for self-harm in prison during COVID-19 were largely consistent with those previously observed.

Several factors could account for the lower incidence of self-harm in COVID-19 year two, including some reductions in restrictions towards the end of this period in some sites, but many of these factors cannot explain why a similar reduction was not evident in the first year of the pandemic. For example, restrictions on movement and socialising may have limited or removed some of the usual risk-factors for self-harm, including limiting the opportunity for disciplinary infractions, and reducing rates of victimisation and bullying during imprisonment.^21^ However, the presence of other risk-factors will have been heightened, including lack of face-to-face social support, with visits from family and friends being restricted, while increased time spent in cells may have been expected to replicate the clear association between self-harm and solitary confinement.^21^ Notably, national trends for prisons in England and Wales suggest that rates of self-harm are returning to levels seen before the pandemic, which were at an all-time high in the twelve months to April 2020.^10^ Whilst this may indicate a change in self-harm behaviour pre-, during and post-pandemic, it is also possible that this reflects a return to pre-pandemic detection rates for self-harm. However, data such as the nature and frequency of routine and enhanced in cell and other checks would be needed to begin to examine this.

Our work is subject to some limitations. First, there are likely to have been differences in the detection and reporting of incidents following the introduction of restrictions, with the figures reported here likely to be an underestimate. For example, introducing restrictions on movement and time spent outside of cells will have allowed fewer opportunities for detection of self-harm by prison staff (e.g. during gymnasium and recreational activities). Second, the relatively transient nature of the prison population, especially in OPDP sites where individuals may transfer in or out of non-OPDP custodial settings, means a relatively small proportion of the sample were eligible for inclusion in one time-period (i.e. must have spent at least one full time-period in an OPDP site), with even fewer being present for the entire three-year period of this study. The movement of people in and out of OPDP services could have biased results. Third, although our findings offer information on the trends of adjudications and self-harm over time, they are not revealing about the underlying mechanisms. For example, it is unclear whether there were changes in mental distress and well-being over time, and whether these changes would account for changes in adjudications and self-harm. Although OPDP sites routinely collect some data on mental distress and prison climate, the collection of these data was not reliably carried out during the pandemic, limiting their usefulness for inclusion in these analyses. Data on pandemic-related measures such as situational impact, social distancing measures, environmental policies and the timing and uptake of vaccinations at individual OPDP sites were also unavailable and may have been useful for inclusion in analyses. Fourth, it would have been helpful to analyse changes in different types of adjudications during the study period, but breaking these down in to, for example, prisoner-on-prisoner and prisoner-on-staff assaults, would have reduced the amount of available data and limited statistical power.

It is important to consider here that a reduction in rates of adjudications and, to a lesser extent, incidents of self-harm does not tell the full story of potential adverse effects of the pandemic on prisoner well-being and distress. A fall in rates of adjudications and self-harm may reflect longer time spent in cells, with limited opportunities to both adjudicate and self-harm, but might also suggest that removing prisoners from the usual prison regime and day-to-day stresses of prison life has some short-term benefits. However, placing people in situations that share overlapping features with solitary confinement is also likely to be associated with considerable long-term harms relating to mental well-being and distress,^23^ and would violate the UK’s commitment to human rights. It is also important to consider that adjudications and self-harm are observational data, and with significantly reduced staffing and increased time spent in cells, these behaviours may have gone unseen by staff and therefore unrecorded, rather than not having happened at all. This would be particularly true for self-harm rather than adjudications, given adjudications tend to be interpersonal in nature. Given the lack of data on underlying well-being and distress, and the well-known adverse effects of solitary confinement, our findings should not be considered supportive of the use of stringent lockdowns either during or outside of emergencies such as pandemics.

Further, our results suggest that younger, White prisoners may represent a particularly high-risk group for relatively sustained levels of adjudications and self-harm relative to other sociodemographic groups. These findings are in stark contrast to findings showing that groups including older people, and people from ethnic, sexual and gender minorities represented the most vulnerable groups during the pandemic,^6,7^ and this discrepancy is worthy of further investigation. These findings may therefore help to identify a particular subset of the OPDP prison population for whom the period of restrictions proved most challenging and should inform future pandemic preparedness. Although our findings can help to inform future policy and decision-making for future events requiring restrictions on movements, social distancing and quarantine of prisoners, it is important to also consider the potential adverse consequences of entering restrictive periods of lockdown, highlighting the need for routine, robust data collection procedures to understand mental health and well-being in prisons.

## Data Availability

The data that support the findings of this study were shared under licence by the Ministry of Justice, UK, and are not publicly available.
